# Impact of Pain Neuroscience Education Program in Community Physiotherapy Context on Pain Perception and Psychosocial Variables Associated with It in Elderly Persons: A Ranzomized Controlled Trial

**DOI:** 10.3390/ijerph191911855

**Published:** 2022-09-20

**Authors:** Juan Vicente-Mampel, Pedro Gargallo, Iker Javier Bautista, Paula Blanco-Gímenez, Nieves de Bernardo Tejedor, Mónica Alonso-Martín, Marta Martínez-Soler, Luis Baraja-Vegas

**Affiliations:** 1 Department of Physiotherapy, Faculty of Medicine and Health Sciences, Catholic University of Valencia, 46001 Valencia, Valencia, Spain; 2 Neuromuscular Physiotherapy, Pain and Therapeutic Exercise Research Group, Catholic University of Valencia, 46900 Torrente, Valencia, Spain

**Keywords:** elderly people, chronic pain, education, quality of life, kinesiophobia, catastrophism

## Abstract

This study investigated the long-term effect (six-months) of a Pain Neuroscience Education (PNE) program on pain perception, quality of life, kinesiophobia and catastrophism in older adults with multimorbidity and chronic pain. Fifty participants (*n* = 50) were randomly assigned to the pain education therapy group (PET; *n* = 24) and control group (CG; *n* = 26). The PET group received six sessions (i.e., once a week, 50 min) about neurophysiology of pain while the CG carried on with their usual life. Perception of pain through the visual analogue scale (VAS), quality of life (EQ-5D questionnaire), kinesiophobia (TSK-11) and catastrophism (PCS) were assessed after six months since the last PNE session. Statistically significant differences on VAS (t_(48)_ = 44, *p* = 0.01, ES = 0.42 [0.13, 0.65]) was found in favor to PET group. No other statistically significant differences were found. This study found that the application of a PNE intervention in an isolated form was able to significantly reduce pain perception with low effect size in the long-term (six months after intervention) in elderly people with chronic pain.

## 1. Introduction

Population aging is recognized as a global phenomenon [1]. According to World Population Prospects 2019, by 2050, one in six people in the world will be above the age of 65 years, up from one in eleven in 2019 [2]. This situation implies a major challenge for public health-care sectors due to human aging and is characterized by a progressive decline in multiple systems [3]. Chronic pain is one of the most common symptoms at old age, suffered by at least 45% of the elders worldwide [4]. It is associated with a worse self-perception of health and it is the leading cause of disability among this population [5]. Although aging and pain are not inherent phenomena, their combination is frequently related through frailty [6], considering pain a predictor of this clinical syndrome [7], which is characterized by an underlying state of decline in reserve and function due to multisystem dysfunction, manifested as a major vulnerability to stress [8]. In addition, chronic pain is also associated with lower mental health quality in terms of depression and anxiety along with lower quality of life and physical function, increasing the chance of being institutionalized [5].

According to the International Association for the Study of Pain (IASP), pain is defined as: “*an unpleasant sensory and emotional experience associated with actual or potential tissue damage, or is described in terms of such damage*” [9]. As a biopsychosocial entity, the treatment of chronic pain should involve a multi-faceted approach that include pharmacologic, surgical, physical as well as psychological interventions. In the last decade, with the growing acceptance of the biopsychosocial model of pain, more attention has been paid to the impact of cognitive and emotional experiences on pain perception, with pain education playing an important role in the management of pain [10]. Some psychosocial factors such as kinesiophobia, fear-avoidance or catastrophizing can increase and perpetuate chronic pain through deconditioning mechanisms of central nervous system [11].

One of the most common approaches to pain education is the pain neuroscience education (PNE) [12]. This therapy is based on cognitive-behavioral interventions that aim to change the maladaptive pain perceptions and beliefs. It is focused on explaining to the patient not only the pathoanatomical factors of pain, but also the complex psychosocial factors such as kinesiophobia or pain catastrophizing that contribute to the pain maintenance [13]. Therefore, the final purpose of PNE is reduce the bodily pain (whether the pain stems from a nociceptive, neuropathic or nociplastic pain disorder, or a combination of them), and consequently, the disability and fear of movement. PNE sessions could be performed in different formats in terms of number of people (i.e., one to one, pairs, small or large groups) and means (i.e., face-to-face, video tutorials, online) [14]. It is suggested that, from all of these formats and teaching methodologies, group lessons are the most appropriate for community and clinical settings since material and personal resources are limited [15]. Since the first study that analyzed the effects of PNE on chronic pain in 2002 [14], several systematic reviews and meta-analysis examined the effects of PNE for patients with musculoskeletal pain, showing no effect or a small to moderate positive effect on pain, pain catastrophizing, kinesiophobia and disability when PNE is performed in isolation or in combination with other therapies [16].

Despite the proven effectiveness of PNE for the management of pain and its psychosocial related factors, there is a lack of information about its effects in the elderly population. The treatment of chronic pain in older adults is complex and usually inappropriate and substantially underestimated for the population with the highest prevalence rate of chronic pain [17]. As a healthcare provider, a physical therapist must provide the best treatment for patients with chronic pain, and therefore, it is urgent to fill the gap of knowledge in this field to cover the needs of the elderly patients with this pathology. However, to date, only the study of Rufa et al., (2019) analyzed the impact of a PNE program in elderly people. After only two sessions of PNE intervention the authors found significant positive improvements in pain disability and fear of movement. It seems that PNE could be an appropriate approach by promoting the individual’s ability to self-manage and face challenges related to their condition. In older people, preserving a certain degree of autonomy is linked to the ability to make decisions and better self-management [18]. Interventions focused on promoting this autonomy can contribute to achieving healthy aging, with a positive impact on frailty [19].

Therefore, in light of the fact that the response in front of PNE programs among young/adult and older adults could differ as a result of these differences, the aim of this study was analyze the effect of a PNE program on pain perception (VAS scale), quality of life (EQ-5D questionnaire) and psychosocial variables related to pain and movement such as kinesiophobia (TSK-11) and catastrophism (PCS) in pre-frail older people (65–90 years old). We had hypothesized that the PNE approach would improve the subjective perception of pain, quality of life, kinesiophobia and catastrophism in older adults with chronic pain.

## 2. Materials and Methods

### 2.1. Study Design

A between groups unifactorial randomized control trial was performed. The sample was composed by Caucasian older adults (>65 years) with multimorbidity. Fifty (*n* = 50) participants were randomly distributed into two groups using a computer-generated random (EPIDAT-Consellería de Sanidade-Servizo Galego de Saúde (sergas.es) permutation procedure (by an independent researcher): pain education therapy group (PET; *n* = 24) and control group (CG; *n* = 26). The study adhered to the CONSORT guidelines [20]. It was conducted in accordance with the Declaration of Helsinki and approval by the local ethics committee of the Catholic University of Valencia (UCV/2019-2020/060). Each included subject signed the written informed consent. The researcher who generated the random allocation sequence also enrolled the participants.

### 2.2. Participants

Participants were recruited from the Senior UNED University for elderly people located in Castellón (Spain). The inclusion criteria were: (1) aged between 65 and 90 years; (2) temporary availability to participate in the study; (3) history of chronic primary musculoskeletal pain of 6 months (according to the IASP in its article entitled “*The IASP classification of chronic pain for ICD-11: primary chronic* pain”) [21] or greater with a value of 4 or higher in the VAS scale (moderate pain at VAS: 31–54 mm; (4) proficiency in literacy and Spanish speaking.

Participants who fulfil any of the following criteria were excluded: (1) severe neurological, vascular or respiratory pathology; (2) systemic or local acute infection or anormal bleeding; (3) complications of other rheumatic diseases; (4) immune suppression or autoimmune illness; (5) cancer-related pain; (6) cognitive impairment (less than 24 in the mini-mental scale examination test); (7) a temporary intake of pain medication during the program or six months before.

Eligibility screening was performed via telephone using a standardized screening form. Before being included in the study, all of the potential participants were comprehensively informed about the study purpose and procedures as well as the benefits, risks, and discomfort that might result from participation. Participants were informed that they could withdraw from the study at any time without any consequence.

### 2.3. Procedures

#### 2.3.1. Experimental Sessions

The program, contents and pictures used for the PNE program were based on the book “Explain Pain” [22]. The therapist individually tailored the educational session basing on this information. The educational program was divided into six topics and was performed in six sessions of 50 min, with a weekly frequency, for six weeks. The information was provided by an expert physiotherapist with more than five years of experience in this topic. Subjects were educated by the same physical therapist for all the sessions. The educational program was carried out in a group way, formed by 24 elders. The therapist who conducted the educational sessions was blinded to the results of the measurements and questionnaires. The main topics addressed during the educational sessions were described in Table 1 (for more information see Supplementary Materials). The educational information was provided through verbal explanations, visual support (power point presentation) and brochures. With the aim of individualizing the information and ensuring the understanding of the concepts, patients were allowed to ask questions during the sessions.

#### 2.3.2. Control Group

Participants in the control group received usual practice in primary care, which included an assessment of VAS, EQ-5D, PCS and TSK-11. All participants were asked to maintain their normal daily routines and eating habits, avoid nutritional supplements or drugs that might affect their body composition, performance and pain, and refrain from beginning new exercise programs or any other type of therapy for the duration of the study.

### 2.4. Outcomes

Pain perception, quality of life as well as psychosocial factors (i.e., kinesiophobia, pain catastrophism) were evaluated in both groups after 6 months of intervention in the same place where the education classes took place. All variables were assessed through a self-administered questionnaire. General sociodemographic and anthropometric data was also collected (age, weight, height, gender, pain medication and comorbidities). In the present study, only one measurement was performed from the different tests used in order to avoid the effects of pain-dependent learning.

#### 2.4.1. Pain Perception

A VAS of 100 mm length was used to assess the current bodily pain perception. This scale started from 0 (i.e., absence of pain) to 100 (i.e., maximum pain) to assess pain intensity [23]. Subjects were asked to mark the point corresponding to their feeling of pain. The patients were asked to indicate the pain they have when the question is performed, therefore at this moment of the day. Previous studies has shown that VAS obtained a high reliability for acute pain (intraclass correlation coefficient [ICC_2_._1_] = 0.97; 95% confidence interval [CI_95%_] of 0.96–0.98) [24].

#### 2.4.2. Quality of Life

The EQ-5D questionnaire was used to assess the quality of life [25]. This tool has been validated for older adults [26] and, in addition, for the Spanish population [27]. It is composed by 5 original dimensions (i.e., mobility, self-care, usual activities, pain/discomfort, and anxiety/depression). The new 5-level Likert-type scales have the following answer options: no problem, slight problems, moderate problems, severe problems, and extreme problems or unable to perform. The instrument also includes VAS on general health [28].

#### 2.4.3. Catastrophism

The Spanish version of the PCS was used in this study to assess pain catastrophism [29]. The PCS is a self-administered scale comprising 13 items. Factor analyses of the PCS have shown that pain catastrophism can be considered a multidimensional construct comprising elements of rumination, magnification and helplessness in which each factor has a value between cero (not at all) to four (all the time) (range 0–52) [30]. Previous studies have shown that the reliability of the PCS was rated as excellent (ICC_2_._1_ = 0.94) [31].

#### 2.4.4. Kinesiophobia

The Spanish version of the TSK scale was used to assess the fear of movement [32]. The scale is composed of eleven items, five of them related with the somatic dimension and six to the activity avoidance. In each item, the subjects must select the degree of agreement with different affirmations selecting from one (strongly disagree) to four (strongly agree) in a Likert scale. The higher score, the higher the fear of movement [33,34].

### 2.5. Statistical Analysis

All variables were expressed as a mean and standard deviation (± SD). The assumptions of normality and homogeneity of variance were evaluated using Shapiro-Wilks and Levene test, respectively. To analyze the differences of the PNE program on dependent variables, an independent *t*-test was performed if the variables were distributed normally. The U Mann-Whitney non-parametric test was performed when the distribution was not normal. The effect size (ES) was calculated through the g Hedges, where <0.20 is considered trivial, 0.20–0.49 low, 0.50–0.80 medium, 0.80–1.20 high and >1.20 very high [35]. The biserial range correlation method was used to calculate the ES, when the variables were not normally distributed. Statistical significance differences were stablished at *p* < 0.05. All of the analyses were performed using a statistical software package (JASP, The Netherlands). This study used a method of convenience sampling similar to other studies such us Sillevis et al. [36] and Rufa et al. [37] with 26 and 25 subjects, respectively, per group.

## 3. Results

### 3.1. Participant Flow and Baseline Characteristics

**Figure 1:**
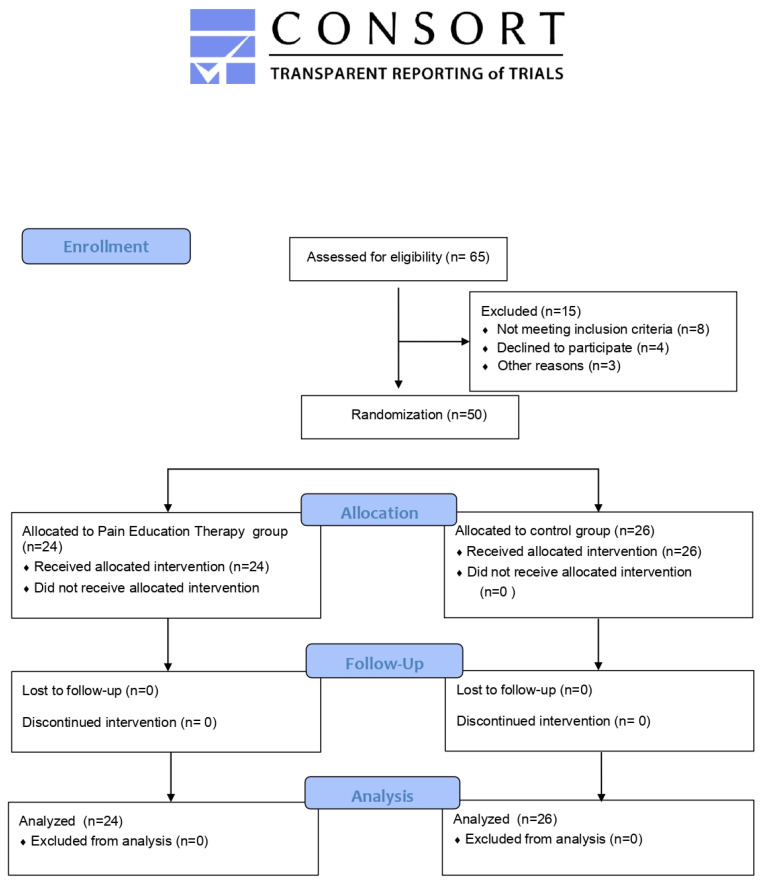
Flow diagram of the study.

Details of the participant flow through the study are displayed in Figure 1. Anthropometric variables corresponding to age, body mass and height of the sample according to each group were: PET group (age = 75.79 ± 5.92 years, body mass = 66.37 ± 14.56 kg and height = 161 ± 6.12 cm) and CG (age = 74.07 ± 6.27 years, body mass = 68.12 ± 8.76 kg and height = 159.23 ± 5.48 cm). No dropouts, missing values and adverse events were recorded. Main characteristics of the sample are displayed in Table 2.

### 3.2. Effects of Intervention

Table 3 summarized the mean and standard deviation on pain, kinesiophobia and catastrophism after PNE intervention in both groups (i.e., PET and CG).

**Figure 2:**
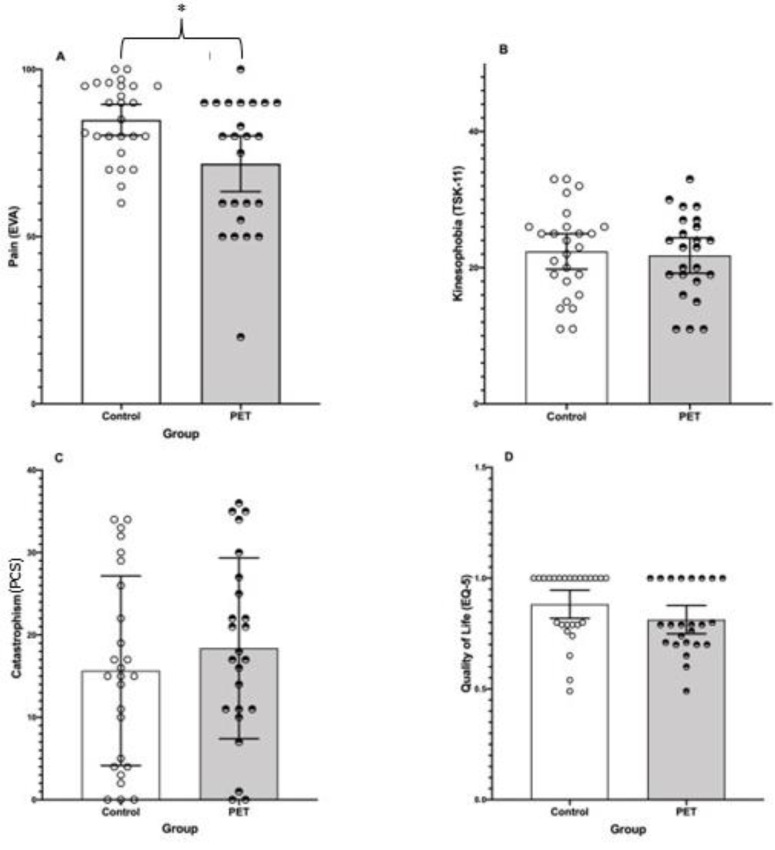
Mean and 95% confidence interval (CI_95%_) of PET and CG groups after the intervention period. White dots represent the individuals of the control group while the black dots represent those of the PET group. (**A**–**D**) graphs: show differences between groups on pain, kinesiophobia, catastrophizing and quality of life, respectively. * means *p* = 0.01.

The independent samples *t*-test did not show statistically significant differences on quality of life (t_(48)_ = 1.59, *p* = 0.12 ES = 0.44 [−0.12, 1.00]), kinesiophobia (t_(48)_ = 0.33, *p* = 0.74, ES = 0.09 [−0.46, 0.65]), and catastrophizing (t_(48)_ = −0.85, *p* = 0.40, ES = −0.24 [−0.79, 0.32]). However, the Mann-Whitney U test showed statistically significant differences on VAS (t_(48)_ = 44, *p* = 0.01, ES = 0.42 [0.13, 0.65]) in favor of the PET group. The mean differences between the PET and CG were −13.10 in favor of the PET group after the intervention period in the pain perception (see Figure 2A). 

## 4. Discussion

The main objective of this study was to analyze the effect of a PNE therapy on pain, quality of life, catastrophism and kinesiophobia in older adults with primary chronic pain according to the IASP. In this sense, the main finding of the present study suggested that the patients undergoing treatment the pain neuroscience educational program presented a decrease in the thresholds of the VAS. Levels were equal in terms of quality of life, catastrophism and kinesiophobia both groups. These findings could reveal that although educational aspects on pain are a cornerstone component of any intervention program, a global therapeutic approach, using other strategies in combination with education on pain or a larger program, should probably be implemented in order to improve clinical manifestations related to chronic pain such as kinesiophobia, catastrophism, and the quality of life.

Our findings partially confirmed our initial hypothesis as we found significant differences between the PET group and the CG at the end of the intervention in the VAS values, achieving lower values the PET group compared to the CG (see Figure 2), but not in the rest of the parameters assessed. Our results are in accordance with previous studies which concluded that the PNE can have a positive effect on pain in patients with musculoskeletal pain [15], with pain reductions between 0.25 to 1.82 points on a 0–10 scale [10]. The PNE addresses beliefs, expectations, and maladaptive behaviors of the individual towards pain and other components such as stress or emotions. Thus, the reduction in pain through the education therapy could be explained from the Neuromatrix Theory since these factors condition the painful experience through the limbic system and other higher centers [38]. However, there are still some controversies about the effectiveness reducing the pain perception of this kind of therapy when it is applied in an isolated way. Amer-Cuenca et al. (2020) and Yildirim et al. (2009) observed a decrease in pain in patients with fibromyalgia and cancer after the application of PNE as isolated treatment [39,40]. However, authors such as Moseley and Butler (2015) did not consider education as the only treatment effective for the reduction in pain and dysfunction [41].

On the other hand, the PNE program did not show any effect on the quality of life in the elderly patients. However, Tavafian et al. (2007) observed an increase in the quality of life after a similar approach [42]. The contrast between the two studies may be due to the differences in the sample population (i.e., 42.9–44.7 years vs. 75.79–74.07), the use of different educational approach (i.e., our study based on pain neurophysiology and the other on pathological aspects), and finally, the tool used to assess the quality of life also differed (i.e., EQ-5D vs. SF-36). In other studies, the decrease in the amount of pain has been related to an increase in quality of life [43,44,45]. Since the underlying mechanisms of both the peripheral and central nervous system associated with pain processing change with age [46] perception and integration of pain feeling may be experienced differently by the elders.

In the present study, no differences were found on kinesiophobia or catastrophism between groups. Despite some authors have previously described that high levels of kinesiophobia could affect quality of life [47,48], our results are in accordance with previous reviews and meta-analysis which the PNE produced statistically non-significant lower TSK values in patients with chronic pain [10]. However, some authors found significant impact of PNE on the improvement of the fear of movement [49], although to date, only the study of Rufa et al. [37] have demonstrated a positive impact of this kind of approach on kinesiophobia in older adults; however, they used the TSK 17 scale in their results [50]. In addition, a key factor that can also explain these differences is the moment in which the assessments were performed (post-intervention in the case of Rufa and collegues vs. after six months). Other studies that have analyzed the effects of PNE in an isolated or combined form with physical therapy have found changes in catastrophism and kinesiophobia [51]. PNE should probably be accompanied by other treatments to achieve a significant effect on these variables, as has been previously reported [49,52].

One of the main issues in the field of the PNE is the heterogeneity of the protocols proposed. Mainly, the therapies applied are a short duration (<three months) interventions carried out in young or adult people with musculoskeletal disorders. In addition, most of the studies performed to date include the combination of PNE with other interventions, but the study of this therapy performed in an isolated way has been less studied [12]. In addition, the number and length of educational sessions, along with the method used to show the pain neurophysiology information to the patient, can also influence the results obtained. In our study the educational program was divided in six topics and was performed in six sessions of 50 min, carried out in a group way. Although it is unclear which protocol could be the most effective and efficient method for delivering PNE, it seems that the individual (one-on-one) modality and longer sessions could be more effective [53], although in some contexts such as in the prevention of cancer, diabetes or hypertension, both modalities have similar effects [54,55,56]. In addition, Gokhale et al., 2020 find similar benefits in both modalities when pain perception was analyzed [57]. It is necessary consider that the group modality could be more cost-effective and efficient than individual education by reducing the time required by health professionals and enhancing the motivation of users.

According to the results of the present investigation, PNE applied in older people with chronic pain could produce a reduction in pain perception. However, this statement should be considered with caution because the minimum clinically important change for pain should be at least 25% [58]. In the present study the percentage of change was not calculated since the measurement was only performed six months after the intervention. However, people’s chronic pain cannot be reduced by the natural course of pain with the passage of time since chronic pain lasting over six months is predominantly somatosensory in nature, not related to acute tissue damage. Hence, the proposed intervention in the present study.

This educational therapy could be a useful tool to use in clinical settings and community contexts as a preventive and treatment measure, which can be applied in an isolated form or in combination with other techniques. Since no equipment and negative side effects are produced, this approach is highly interesting to be applied in the elderly population. If the older adults present lower perception of their pain, the morbidity could be reduced by producing an increase in the level of physical activity. Through education, it is possible to modify the personal habits that older people have, in addition to their beliefs. To achieve an effective modification of the aforementioned components through education, it would be convenient to observe its effects when applying it continuously in a longer period, taking into account also the influence of some demographic and characteristics of the person such as the social status, body mass index, education level, medication, level of disability, etc. [59].

The present investigation has some limitations that must be considered when attempting to draw evidence-based conclusions. The results reported in this experiment are specific to older adults with multimorbidity and, therefore, should not be extrapolated to other populations. The absence of pre-intervention measurements makes it difficult to observe the real effect in the experimental group, although the design of the present study is justified due to the existence of a possible learning effect in the parameters evaluated [60]. The levels of physical activity, depression, anxiety and other physical and psychosocial variables that could modified the symptoms were not controlled. Future research should be conducted to determine which PNE program could be more effective in terms of modality, duration, and types of sessions for improving pain and its related psychological aspects in older adults in different settings.

## 5. Conclusions

This study found that the application of a PNE intervention in an isolated form was able to significantly reduce the pain perception with low effect size in the long-term (i.e., 6 months after intervention) in elderly people with chronic pain. Although further randomized control trials are needed to confirm our results, PNE could be considered a suitable treatment to reduce pain at old ages.

## Figures and Tables

**Table 1:** Main topics of the Pain Neuroscience Education protocol.

Topic	Type of Topic	Week
Topic 1	Acute vs. chronic Pain	Week 1
Topic 2	Acute Pain Physiology: nociception process	Week 2
Topic 3	Pain Chronification Processes I: peripheral sensitization	Week 3
Topic 4	Pain Chronification Processes II: central sensitization	Week 4
Topic 5	Chronic Pain Neurophysiology: neuromatrix theory	Week 5
Topic 6	Pain Dimensions: emotional, psychological, and social factors	Week 6

**Table 2:** Anthropometric and sociodemographic characteristics of the sample.

	PET (*n* = 24)	CG (*n* = 26)
Age (years)	75.79 ± 5.92	74.07 ± 6.27
Body Mass (kg)	66.37 ± 14.56	68.12 ± 8.76
Height (cm)	161 ± 6.12	159.23 ± 5.48
Participants without co-morbidities or minor disorders otherwise from pain	4	5
Participants with co-morbidities	20	21
Co-morbidities, % (*n*):		
Diabetes	6 (23%)	4 (15%)
Hypertension	9 (34%)	8 (30%)
Hypercholesterolemia	15 (57%)	10 (38%)
Arthrosis	2 (7%)	1 (3%)
Others (prostatitis, rheumatoid arthritis, hypothyroidism, lymphoproliferative syndrome, heart disease, breast carcinoma)	1 (3%)	7 (26%)
Pain medication use:		
Any pain medication use	23 (96%)	22 (84%)
Paracetamol	1	2
Ibuprofen	0	1
Other Analgesics	0	1

Note: Regarding the pain medication, participants should continue with their pharmacological treatment regimen, being for all of them a medication that usually take in their daily life during the last year.

**Table 3:** Summary of descriptive statistics (mean and standard deviation) of outcomes measured in both groups (PET and CG) after six months of intervention.

	*n* (F/M)	Pain (VAS) ^#^	Quality of Life (EQ-5D)	Kinesiophobia (TSK-11)	Catastrophism (PSC)
PET	24 (14/12)	71.79 (19.72) *	0.81 (0.15)	21.79 (6.15)	18.38 (10.97)
CG	26 (13/13)	84.89 (11.49)	0.88 (0.16)	22.39 (6.44)	15.65 (11.51)

Note: PET: pain education therapy group; CG: control group; F: female; M: male; TSK-11: Tampa Scale of Kinesiophobia 11; PCS: Pain Catastrophizing Scale. ^#^ Pain variable (VAS) was the only one not normally distributed (W = 0.892, *p* = 0.015). * *p* < 0.05, significant difference with respect to the CG.

## Data Availability

Data supporting results could by available by sending and e-mail to the corresponding author.

## Supplementary Materials

The following supporting information can be downloaded at: https://www.mdpi.com/article/10.3390/ijerph191911855/s1, Curriculum content of the Pain Neuroscience Education Program.
