# Supplementary material for: Impact of Pain Neuroscience Education Program in Community Physiotherapy Context on Pain Perception and Psychosocial Variables Associated with It in Elderly Persons: A Ranzomized Controlled Trial
Source: Int J Environ Res Public Health. 2022 Sep 20;19(19):11855. doi: 10.3390/ijerph191911855 (PMC9565887; doi:10.3390/ijerph191911855)
Supplement: Supplementary file 1 [file ijerph-19-11855-s001.zip › ijerph-1866403-supplementary.pdf]

WEEK / SESSION	MAIN TOPIC	CONTENTS	GOALS	REINFORCEMENT	METHODS	TIME
1	**Acute vs. chronic pain differentiation.**	- Pain definition.<br>- Nervous system as an alarm system.<br>- Nociception vs. pain.<br>- Sensory stimuli: mechanical, thermal, and chemical.<br>- Differences between acute and chronic pain.	- Exploring patient beliefs about pain.<br>- First approach to nervous system knowledge.<br>- Understanding pain as an alarm system.<br>- Demystify chronic pain.<br>- Self-questioning of pain beliefs and behaviors.	- Pain as an alarm system.<br>- Chronic pain does not mean forever.<br>- Introspection about personal beliefs and pain.		60 minutes
2	**Acute pain physiology.**	- Nociception process: stimuli transduction, transmission, and modulation<br>- Top-down inhibitory systems<br>- The relevance in motion and behavioral<br>- Relationship between pain and tissue damage	- Learn about how a stimulus can produce nociception.<br>- Understand how nociception can be modulated by nervous system.<br>- Think about pain and movement.	- Nociception and pain are not the same.<br>- Pain is rarely related to tissue damage.	- Metaphors<br>- Drawings and diagrams made by the researcher.<br>-Illustrations, definitions and diagrams extracted from ***"Explain Pain".***	60 minutes
3	**Pain chronification processes I: peripheral sensitization.**	- Introduction to sensitization.<br>- Hyperexcitability of nociceptive pathways.<br>- Hyperalgesia<br>- Peripheral sensitization as an adaptive protective process.	- Understanding the plasticity of the nervous system.<br>- Why certain areas may be more reactive to nociception.<br>- Learning about inflammation and pain.	- The same stimulus can be more painful in the presence of sensitization.<br>- Peripheral sensitization and hyperalgesia are protectives mechanisms.		60 minutes

Curriculum for 360 minutes of Pain Neuroscience Education, in a group session, for elderly patients with chronic musculoskeletal pain, based on a previous guideline (Moseley, G.L.; Butler, D.S. Explain Pain Supercharged, 1st ed.; Noigroup Publications: Adelaide, Australia, 2017; p. 244)

WEEK / SESSION	MAIN TOPIC	CONTENTS	GOALS	REINFORCEMENT		TIME
4	**Pain Chronification Processes II: central sensitization.**	- Top-down inhibitory systems.<br>- Wind-up phenomenon and long-term potentiation.<br>- Allodynia and hyperalgesia concepts.<br>- Maladaptive consequences.	- Learn the role of the central nervous system in pain modulation.<br>- Learn to identify the role and signs of allodynia and hyperalgesia.	- Decreased descending inhibition may cause more pain in the absence of tissue damage.<br>- In presence of allodynia, non-painful stimuli can pro pain		60 minutes
5	**Chronic Pain Neurophysiology: Neuromatrix theory.**	- Neuromatrix<br>- Role of the limbic system on pain.<br>- Pain pathways on brain.<br>- First approach to cognitive and emotional parts of pain.	- Understand individual and subjective features of pain.<br>- Identify the different pathways of pain in the brain.<br>- Understand the role of limbic system on pain.<br>Think about threat perception and limbic system responses.	Pain does not occur on tissues.	- Metaphors<br>- Drawings and diagrams made by the researcher<br>- Illustrations, definitions and diagrams extracted from ***"Explain Pain".***	60 minutes
6	**Pain Dimensions: emotional, psychological, and social factors.**	- Experience, beliefs and emotions on pain perception.<br>- Protectometer<br>- Context influence.<br>- Maladaptive behaviors on pain.<br>- Fear-avoidance, hypervigilance, catastrophizing and kinesiophobia.	- Think about personal experiences and injury.<br>- Understand the influence of context and emotions on perceived pain.<br>- Think about personal behaviors that can be perpetuating pain.<br>- Design and learn strategies of self-efficacy on pain.<br>- Understand the role of attention and hypervigilance on pain.	- Emotions, beliefs, and context are part of pain experience.<br>- Maladaptive behaviors related to pain must be identified.<br>- Self-efficacy on pain.<br>- Concepts related to movement and pain and fear-avoidance behaviors.		60 minutes

Curriculum for 360 minutes of Pain Neuroscience Education, in a group session, for elderly patients with chronic musculoskeletal pain, based on a previous guideline (Moseley, G.L.; Butler, D.S. Explain Pain Supercharged, 1st ed.; Noigroup Publications: Adelaide, Australia, 2017; p. 244)
